# Being torn by inevitable moral dilemma: experiences of ICU nurses

**DOI:** 10.1186/s12910-021-00727-y

**Published:** 2021-11-30

**Authors:** Neda Asadi, Zahra Royani, Mahbubeh Maazallahi, Fatemeh Salmani

**Affiliations:** 1 Nursing Research Center, Kerman University of Medical Sciences, Kerman, Iran; 2 Ph.D Candidate in Nursing, Nursing and Midwifery School, Golestan University of Medical Sciences, Gorgan, Iran; 3 Nursing and Midwifery Sciences Development Research Center, Najafabad Branch, Islamic Azad University, Najafabad, Iran

**Keywords:** Intensive care unit, Nurses, Ethical decision-making, Content analysis

## Abstract

**Background:**

Ethical decision-making of nurses could affect patients’ recovery and also decrease medical costs. To make ethical decisions, ICU nurses experience complicated ethical conflicts. Considering the multi-dimensional process of ethical decision-making, the present study was conducted to describe the experiences of ICU nurses regarding ethical decision making.

**Method:**

The present research is a qualitative study with conventional content analysis approach that was done in 2020. Fourteen ICU nurses were interviewed using a semi-structured in-depth interview method. The interviews were recorded, transcribed verbatim, and analyzed using Granheim and Landman approach.

**Results:**

Being torn by inevitable moral dilemma was defined as the main category which contained the categories of conflict with professional self, feeling squeezed between self-authority and demands of others, and Surrounded by organizational limitations.

**Conclusions:**

Results of the present study showed that moral dilemma is a situation in which the nurses are forced to choose between two options based on their equipment and facilities. In these situations, the nurses would hesitate between their internal conflicts and confusion between choosing their own benefits and patients’ benefits. To prevent ethical challenges in decision making, it is necessary to educate healthcare team about ethical dilemmas and empower the personnel for encountering ethical challenges.

## Background

Ethical decision-making is a rational process which is considered as making the best ethical decision through systematic argument when contradictory choices are available [1]. Practically, intensive care units nurses usually face situations that would require them to make fast decisions or choose from uncertain choices [2]. Those, along with other healthcare professionals, play a critical role in ethical decision making during the care process. Ethical decisions could affect patients’ recovery and also decrease medical costs while irrational decisions with weak timing could lead to decreased quality of care and delay in the process of patients’ recovery [3]. Therefore, recognizing the best ethical act and making correct ethical decisions requires professional nurses who are committed to their profession. Meanwhile, intensive care units are considered as the most critical wards of the hospitals and ICU nurses might experience complicated ethical conflicts in the face of life threatening factors, unusual condition of the patients regarding their life and death, expensive equipment, aggressive and ineffective treatments (unnecessary tests), false hope for the patient's family, a lack of control on tasks related to job descriptions, and continued resuscitation of patients with end-stage disease [4].

Nurses should resolve these ethical problems in the practice; for this purpose, they should search for appropriate secondary options to make a cautious ethical decision. To make ethical decisions nurses are faced with a wide range of effective and determinant factors [5]; individual factors such as values, beliefs, experiences, knowledge, and skills and also background factors such as ethical awareness, observation and judgment could be effective on the ethical behavior and decisions of the nurses. Nurses would use factors such as educational, religious beliefs, their intuition and feelings, guidelines, criteria, colleagues’ support and possible outcomes to justify their decisions. Also, the patients’ characteristics are effective on nurses’ decisions [6]. Understanding the effective factors on making ethical decisions is of great importance [7]. Considering that Iran is a multi-ethnic community with various religions and ethnicities, each has its specific customs, language and identity, the importance of culture would become more significant [8] and culture is also an essential factor in making ethical decisions [9]. The presence of these factors would lead to complication and uncertainty at the time of decision -making [5]. On the other hand, along with the development of their professional role, their responsibility in the field of decision making has also become greater [10]. However, despite ethical advances in the field of health care, there is limited information about making ethical decisions in Iran, and there is not sufficient information about the ability of nurses in following up their decisions and their outcomes and also the effect of the clinical conditions on their decisions [6].

Qualitative research methods have been based on common intellectual foundations and these methods, with an insight from the inside, have been focused on the manner that individuals would perceive the world around them and are looking to understand social realities, emotions, behaviors, and experiences from the viewpoint of activists, cultures and groups [11]. Considering the multi-dimensional process of ethical decision-making in nurses, it is only possible to achieve through qualitative approach and in-depth studying of the nurses’ lived experiences who are constantly faced with various challenges. On the other hand, literature review has shown that, despite the studies that have been conducted on ethical decision making, few qualitative studies have investigated the experiences of ICU nurses in Iran and there is a gap in the knowledge of this field. Therefore, the present study was conducted to describe the experiences of ICU nurses regarding ethical decision making.

## Methods

### Design

The present research is a qualitative study that has been conducted using conventional content analysis method. In this method, a phenomena would be described by analyzing written, spoken or visual messages. Summarizing and categorizing raw data would be performed by inferences and interpretation and categories and their names would be extracted from the context of the data using in-depth analysis [12]. This study presents the ethical dilemma experiences of nurses in Iran.

### Sample and setting

In Iranian health context here, most nurses had a bachelor's degree and specialized training in intensive care units who worked in the Level 3 Intensive Care Units of three governmental hospitals in Kerman, southeast Iran. These hospitals have the highest rate of patient admission to intensive care units with various diagnoses. The nurse-to-bed ratio in intensive care units is 1:2.

Participants were selected using purposeful sampling method from the ICU nursed who had rich experiences regarding the subject of the study and were willing to participate in the study. Sampling was purposively done with maximum variation in terms of participants’ gender, educational level, and work experience. Sampling was initially purposive, and then a theoretical sampling method was applied until data saturation was reached.

### Data collection

The study was conducted between July 2020 and October 2020. Data were collected through in-depth individual semi-structured interviews. Participants were ICU nurses with working experience of more than 5 years. In this study, for 14 nurses in-depth semi-structured interviews were conducted. The time and place of the interviews were determined by the participants. All of the interviews, which lasted between 30 and 60 min, were conducted by one researcher. The interviews were started with the general question of “Please talk about an ethically difficult care situation that you have faced with that”. The interviews continued with the question “How did it affect your clinical decision? Describe your experiences”. The subsequent questions such as ‘How did you feel then?” were guided based on the information provided by the participant and on the researcher's desire to clarify relevant issues.

### Data analysis

Data were analyzed through five-step conventional content analysis method proposed by Graneheim and Lundman [13]. In the first step, each interview was transcribed word by word. In the second step, the interview transcript reviewed several times to obtain a sense of the whole. In the third step, each interview transcript was considered as the unit of analysis, then meaning units were identified and coded. The first author analyzed the total data, while the second one analyzed half of the textual data. Two authors then compared the codes, and revised minor disagreements after discussion. In the fourth step, codes grouped into subcategories according to their conceptual similarities and differences. In the fifth step, subcategories compared with each other and the latent data content identified and presented as the categories. Eventually, three categories were merged into one the main category based on the latent data content.

The final three categories and one main category were examined by all authors to ensure a clear difference between categories and subcategories and fit the data within each category.

### Trustworthiness

To determine the accuracy of the data, four criteria of credibility, dependability, confirmability and transferability were used [13]. Credibility was established using member and peer-checking, prolonged engagement, and maximum variance of participants’ selection. For instance, for member-checking, a brief report of the findings was given to two clinical nurses, whom they were asked to reflect their experiences and perspectives to the analysis report for researcher assurance. For peer-checking, two qualitative researchers approved the primary codes and categorizing process. Transferability achieved via the provision of a rich description of data collection, analysis processes and findings to allow the readers to match the findings with their contexts.

### Ethical consideration

The Research Council and the Ethics Committee affiliated to Kerman University of Medical Sciences approved the study proposal with code IR.KMU.REC.1399.059, and the number 97000920. Participants were invited to the study by the researcher. Participants were given the opportunity to think then they enter the study after the call. The aims of the study were explained in detail to the participants. The following information was given to the participants: the voluntary nature of the participation, their right to privacy, anonymity and confidentiality as well as right to withdraw from the study at any time without any penalty. The participants then confirmed an informed consent form.

## Results

In this study 14 ICU nurses were participated; 9 women and 5 men with a mean age of 34 ± 6.46 years and a mean working experience of 7 ± 2.31 years. 10 participants had a bachelor’s degree and 4 had a master’s degree (Table 1).

**Table 1:** The demographic characteristics of ICU nurses who participated in the study

Gender	Women	9
	Men	5
Age	Mean ± SD	34 ± 6.46
Working experience (years)	Mean ± SD	7 ± 2.31
Educational level	Bachelor	10
	Master	4

Participants expressed their perceived ethical conflicts in the intensive care units. Being torn by inevitable moral dilemma was defined as the main category which contained the categories of conflict with professional self, feeling squeezed between self-authority and demands of others, and Surrounded by organizational limitations (Table 2).

**Table 2:** Main category, categories and subcategories

Subcategories	Categories	Main category
Dilemma in performing their role	Conflict with professional self	Being torn by inevitable moral dilemma
Dilemma in choosing between the benefits of the patients or the nurses		
Conflicts with co-workers due to limited power	Feeling squeezed between self-authority and demands of others	
Conflicts with patients’ companions		
Lack of personnel for providing care to the patients	Surrounded by organizational limitations	
Shortage in up-to-date equipment		

Conflict with professional self: reviewing the viewpoints of the participants showed that most of them had experienced “dilemma in performing their role” and “dilemma in choosing between the benefits of the patients or the nurses”. Regarding “dilemma in performing their role” participants stated that the assigned duties were unspecialized and time-consuming, would decrease their work force and also would lead to dissatisfaction with the performed cares. Incompatibility between professional duties and organization’s expectations had caused them to experience a sense of dilemma and dissatisfaction with performing their role, and most of the participants considered as a cause for conflict with professional roles. In this regard, participant No. 3 stated: “Time-consuming and repetitive writings are not really my duty. I do not know if I am the ward’s secretary or a nurse. I am busy with the writings and documentation until the last second of my shift. Is documentation my main duty or taking care of patients? I do not know it myself either” (P.3). Considering the Iranian culture and religion, another participant believed that respecting religious principles is necessary in providing care and stated that performing non-nursing duties would prevent them from considering this aspect of care which would lead to a long-term conflict for the nurses: “I still remember the day that I could not respect the privacy of my dying patient. Failure to respect this (religious principles) would hurt me. I was constantly entangled in phone follow-ups. Documentations had consumed all of my time” (P.4).

“Choosing between the benefits of the patients or nurses” indicated participants’ experiences regarding being at a crossroads while performing care for the patients. Participants stated that, in some care situations such as the physician's unavailability, her/his tardiness in arriving at the patient's bedside, and the failure of invasive interventions, They did not know how to protect the benefits of the patients and the benefits of themselves, simultaneously; in a way that, if they would have any protest against the existing conditions, they might endanger their occupation. Therefore, they are entangled in a constant battle between staying silent and protesting, while, they consider the patients’ concerns their own concerns. In this regard one of the participants said: “Although I know that I am not doing the right thing, as long as it would not hurt my patients, I would not protest. Because it might end up hurting myself” (P.1).

“In many situations, I would be forced to choose between myself and my patient. But it is not always like this. Being put in this situation is really tiring…. I had to call, remind and follow up all the time to find the doctor. When he arrived, he did not treat well. Well finally he was the hospital CEO.” (P.9).

In this regard, by mentioning the inevitability of choosing between two options and having hesitate in choosing, one of the participants said: “For example I knew that my patient had coagulation problem. So the patient should not be intubated now, but the physician did not accept. I really don’t know if I should protest or not? If you protest too much, you would be questioned yourself” (P.3).

Feeling squeezed between self-authority and demands of others: One of the matters that could be concluded from the participants’ statements is that, in providing care for the patients, nurses usually face conflicts with their colleagues including physicians, nurses, nursing mangers, and also patients’ companions. It seems that in situations when the possibility of trauma to the patient is predictable or the procedure would be conducted without patient’s consent, nurses would experience moral conflicts with their colleagues. One of the participants said: “It has happened many time that the physicians wanted to perform a procedure without patient’s consent. I have not accepted and warned the physician. But sometimes you don’t know what to do; should you say something or not…” (P.10).

Another nurse mentioned the patriarch of physicians as the reason for these experiences, and while objecting this issue, stated: “While the nurse has a different understanding of the patients’ needs, should perform the care program under the physicians’ order; because they are the head managers and they reign the hospital… This conflict would put more physical and mental pressure on us” (P.2).

“The physicians’ manner is more similar to managers, rather than being a colleague. For example the patient did not have a stable situation, his/her saturation was dropped, but the physician insisted on taking him/her to bronchoscopy. Well, you don’t know what to do here… but you should follow the physician’s order” (P.10).

Participants believed that their spiritual beliefs were effective on patients’ care issues and on the other hand, had an internal conflict for providing correct information to the family patient.

One of the participants said: “Euthanasia is not accepted in our culture and we are not allowed to turn off the devices… They (patient’s companions) are relying on God until the last moment and we cannot disappoint them… Making someone disappointed is considered a great sin in our culture. I have sometimes, even given false hope to them and asked them to rely on God… In these situations, I also feel a great pressure whether to tell the truth or not” (P.7).

Surrounded by organizational limitations: Nurses’ experiences have also revealed Surrounded by organizational limitations. In explaining their experiences, most of the participants have mentioned lack of personnel for providing care to the patients and also shortage in up-to-date facilities and equipment that would lead to unfair provision of care to the patients. This could be effective on occurrence of conflicts in the nurses.

In this regard, one of the nurses said: “Sometimes, it is really out of our hands… the government should put out a recruitment notice to hire more personnel. All of these would affect the ability and the quality of the provided care. It could increase patients’ suffering. Increase their complications…” (P.3).

Another nurse mentioned the effect of shortage in personnel and ignoring some of the patients’ needs and said: “Unfortunately, I am aware that, morally, I have to do both of the procedures for the patient, but I have to perform only one of them, I cannot find any other way. The ratio of beds to nurses is not standard and the duties are so heavy” (P.7).

Furthermore, nurses also mentioned shortage in the facilities and equipment. For example, one of the participants said: “The number of beds is not enough for trauma patients. The patient should be hospitalized in the ICU but they only put an extra bed here; it would not be possible to provide special care for the patient” (P.5).

Some of the participants objected to this issue and stated that it would make them confuse for choosing the manner of providing care. “I have even stated my objection to the supervisor but he/she said that this is it, all the hospitals are facing shortage… while all of them (care procedures) are important equally and ignoring any of them is unethical. But when the patient’s drugs could not be found or there is no HME filter to be replaced in-time, you could not do anything.” (P.3).

## Discussion

Results of the present study showed that moral dilemma is a situation in which the nurses are forced to choose between two options based on their equipment and facilities. In these situations, the nurses would hesitate between their internal conflicts and confusion between choosing their own benefits and patients’ benefits. In these situations, nurses would also feel squeezed between self-authority and demands of others and they would try to resolve these conflicts. At these crossroads, the nurses are faced with shortage in equipment and facilities and even human resources which would cause them face more conflicts in making ethical decisions making.

Results of the present study were in line with the results of a systematic review study titled ethical challenges in nursing that was conducted by Heydari and Meshkin [14]. They mentioned ethical dilemmas in their study that was explained as nurses being forced to choose between two options while they were not sure about their correctness for ethical decision making. The options had no certainty and led to choosing between two equally undesirable options [14].

Also, results of the present study showed that nurses experienced conflict with professional self in ethical decision making while performing cares and decision making, they are hesitant between their own benefits and the patients’ benefits. They have negative beliefs regarding their abilities in performing care for patients in a way that it would cause problems for the nurses in ethical decision making. Taking blood samples, writing various reports, and preparing the patient's medical record, for example, are some of the activities that waste the nurse's time and reduce the time that should be devoted to patient care, making the nurse feel guilty. The nurse has no right to object due to a lack of human resource in the ward because he or she may be reprimanded or even fired for protesting against non-nursing activities. According to this finding, high-ranking hospital managers may expect them to perform tasks that are part of their job descriptions because job descriptions for all medical and paramedical categories have been announced to hospitals by universities, and everyone has the right to refuse tasks that are not part of their job descriptions. Ranjbaran et al. [15] and Nolan et al. [16] showed that personal factors (including culture, society, religion, beliefs, values of the society, nursing personnel experiences, nurses’ perception of their own role, and recent education of the personnel) have an important role in ethical decision making and if, personal factors are not evolved sufficiently, the individual would face internal conflicts at the time of decision making which would cause challenges in the way of ethical decision making. In the present study also, nurses were faced with conflict and hesitation when choosing between their own benefits and patients’ benefits.

Juujärvi et al. [17] in their study mentioned the problem of temptation of the care team, the temptation for encountering one’s needs and demands, achieving resources or advancing personal benefits through intangible, unethical, unfair or ungrateful behaviors to address internal conflicts and these personal demands would usually cause ethical challenges in the path of clinical decision making. Also, responding to controversial demands of would make them face consequences and they would be forced to choose who to help or whose demand to respond [17]. In line with the present study, Ebrahimi et al. [6] concluded that one of the most important ethical conflicts in clinical decision making is to prevent harm to the patient or to the nurse. They stated that most of the times, nurses, to prevent harm to themselves, would not speak the truth and give fake hope to the patients and these strategies would cause harm to the patients [18].

Also in the present study, in the category of feeling squeezed between self-authority and demands of others, it was revealed that sometimes nurses would face problems in their relationships with other nurses, physicians and patients’ companions that would put them at an ethical crossroad and many of these conflicts were caused due to lack of authority of nurses in performing care for the patients. Participants’ statements indicated that nurses confronted with a doctor's unconditional acceptance, did not participate in healthcare decisions, ignored unintentional errors of novice colleagues, and gave false hope to the patient's companion. As a result, it is recommended that in clinical care wards where a physician resides, a decision be made with the participation of the nurse and other healthcare providers to avoid future problems for the nurse and other healthcare team members.

In line with the present study, Redman and Fry showed that ethical conflicts are the inevitable part of clinical activities. They believed that conflicts are caused due to beliefs, duties, principles and theories in which any part of ethical conflicts would be defendable. Although nurses are directly responsible for taking care supporting the patients, they usually lack the authority for decision making and, especially due to experiencing conflicts, they become vulnerable. By reviewing 23 articles about ethical conflicts in nursing, they stated that nurses believed most of the ethical conflicts (75% of the nurses) were related to disagreement with the quality of medical services that were provided for the patients. Mostly, in ethical principles, conflicts occur between profitability principle and innocuous principle. Also in their study that was about determining all types of ethical conflicts in nursing, the relation between conflicts and variables in the working, education and demographic environments, they showed that disagreement with medical intervention (61%) was the most significant clinical example for occurrence of conflicts in nurses [19].

Also Davis [20] in their study showed that, considering that ethical decision making is always associated with stress, independence would allow the healthcare and medical teams to make better decisions making for the patients. They mentioned that the duty of the healthcare team, as the defenders of the patients, is to make sure that conscious decisions for the patients has been made by gaining sufficient information including the possible risks, benefits and complications. Therefore, in most cases, interpersonal conflicts and lack of authority for performing care for the patients would cause ethical challenges for clinical decisions making [20]. In line with the category of feeling squeezed between self-authority and demands of others in the present study, Haahr et al. [21] in their review study revealed a disagreement between the members of the healthcare team. They stated that the difference between skill and responsibility was meant that nurses could have disagreement with physicians. Most of the times, both groups had different strategies for treatment and disagreement within the team about the level of treatment might cause ethical conflicts. In the present study also, nurses faced difficulty in decision making due to the conflicts between nurses and physicians [21]. However, if ethical conflicts in performing care for the patients would not be determined and resolved, it could seriously the ability of the caregiver and also the quality and quantity of the provided care for the patients and consequently, the patients would be deprived from receiving quality care.

In the present study, the category of Surrounded by organizational limitations, mentioned the existing shortage in human resources, facilities and equipment which could be effective on ethical decisions making. For example, if all of the beds in intensive care units are full and there is no mechanical ventilation system, there will be always a conflict over which patient has the highest priority for connection to the mechanical ventilation system or discharge from the intensive care unit. It will be extremely difficult for the nurse or health care team to clinical decision making in these cases and they always think whether the outcome of this decision will harm the patient or not. According to this finding, organizational and nurse managers should try to eliminate equipment and personnel shortages so that nurses and the rest of the healthcare team can focus on providing the best care and making the best decisions for the patient.

Haahr et al. [21] in their study also mentioned the effect of heavy workload on the quality of care. They stated that increased workload of the nurses and shortage in the nursing personnel would affect the quality of their work, decrease their ethical sensitivity, and eventually their ethical decisions making [21]. This study showed that ethical dilemmas can cause many problems in the decision-making process that have not been addressed in other studies.

## Limitations

This study accurately reflects the experiences of ICU nurses in terms of ethical decision making and challenges, but the most significant limitation, as with other qualitative studies, is the lack of generalizability of the study findings. Given that ethical decision-making is a cultural concept, researchers suggest that more research in this area be conducted in other medical settings. Another Limitations of the study included the lack of sufficient opportunity for nurses to be interviewed, which was chosen at the time of the interview in coordination with them.

## Conclusions

Results of the present study showed that ethical decision making is a difficult and complicated process and various personal, interpersonal and organizational factors would affect it. To prevent ethical challenges in decision making, it is necessary to create organizational culture, educate the entire medical and healthcare team about ethical dilemmas and empower the personnel for encountering ethical challenges. It is also suggested that the issues of ethical decision-making be included in the curriculum of nurses and physicians so that this healthcare professional can more easily make decisions when face of ethically difficult situations.

## Data Availability

The datasets used during the current study are available from the corresponding author on reasonable request.

## Abbreviation

ICU: Intensive care unit
